# Sunk cost sensitivity during change-of-mind decisions is informed by both the spent and remaining costs

**DOI:** 10.1038/s42003-022-04235-6

**Published:** 2022-12-07

**Authors:** A. David Redish, Samantha V. Abram, Paul J. Cunningham, Anneke A. Duin, Romain Durand-de Cuttoli, Rebecca Kazinka, Adrina Kocharian, Angus W. MacDonald, Brandy Schmidt, Neil Schmitzer-Torbert, Mark J. Thomas, Brian M. Sweis

**Affiliations:** 1 Department of Neuroscience, University of Minnesota, Minneapolis, MN 55455 USA; 2 San Francisco Veterans Affairs Medical Center, San Francisco, CA 94121 USA; 3 Nash Family Department of Neuroscience, Icahn School of Medicine at Mount Sinai, New York, NY 10029 USA; 4 Department of Psychiatry and Behavioral Sciences, University of Minnesota, Minneapolis, MN 55454 USA; 5 Graduate Program in Neuroscience and Medical Scientist Training Program, University of Minnesota, Minneapolis, MN 55455 USA; 6 Department of Psychology, University of Minnesota, Minneapolis, MN 55455 USA; 7 Department of Psychology, Wabash College, Crawfordsville, IN 47933 USA; 8 Department of Psychiatry, Icahn School of Medicine at Mount Sinai, New York, NY 10029 USA; 9 Present Address: Epic Systems, 1979 Milky Way, Verona, WI 53593 USA

**Keywords:** Computational neuroscience, Decision

## Abstract

Sunk cost sensitivity describes escalating decision commitment with increased spent resources. On neuroeconomic foraging tasks, mice, rats, and humans show similar escalations from sunk costs while quitting an ongoing countdown to reward. In a new analysis taken across computationally parallel foraging tasks across species and laboratories, we find that these behaviors primarily occur on choices that are economically inconsistent with the subject’s other choices, and that they reflect not only the time spent, but also the time remaining, suggesting that these are change-of-mind re-evaluation processes. Using a recently proposed change-of-mind drift-diffusion model, we find that the sunk cost sensitivity in this model arises from decision-processes that directly take into account the time spent (costs sunk). Applying these new insights to experimental data, we find that sensitivity to sunk costs during re-evaluation decisions depends on the information provided to the subject about the time spent and the time remaining.

## Abstract (web summary)

Computationally parallel ‘change-of-mind’ tasks in mice, rats and humans are analysed and demonstrate that sensitivity to sunk costs during re-evaluation depends on the awareness of time spent and remaining.

## Introduction

Although, logically, decisions should be made based on future expectations, many foraging decisions show an escalation of continued pursuit of reward as a function of spent resources (sunk costs)^1–6^. Although sunk cost sensitivity has been observed in non-human animals as well as in humans^7–13^, the human and non-human experiments were done on computationally different tasks. Translating decision processes between species depends on the computational alignment between how each subject makes decisions on a given task^14^.

In a suite of previous studies, we developed a family of new computationally-aligned foraging tasks in which subjects made *accept/skip* decisions for consumable rewards. In these tasks, subjects face a decision of whether to wait out a fully-signaled delay before getting the reward or leaving that offer and trying their luck at a subsequent offer for a different reward. Subjects have a limited time to gather these rewards within a session, making time a limited resource, and this an economic task. Multiple variants of these tasks exist, including ones in which the consumable reward is food (*Restaurant Row* [mice, rats]^15–22^, *Candy Row* [humans]^23^) or videos (*Web-Surf* [humans]^19,24,25^, *Movie Row* [humans]^23^). In the Restaurant Row task, mice or rats physically run around an environment, encountering four different “restaurants” cyclically, each providing a different flavor of food reward. In the Web*-*Surf and Movie Row tasks, humans encounter four video galleries cyclically, each providing either still photographs^24^ or short videos^23–26^. The Web*-*Surf results have been replicated both in-person^23–25^ and online^23,26^, under multiple variations. In the Candy Row task, humans are given the opportunity to receive candy/snacks from four dispensers cyclically^23^. In all of these tasks, the delay to be waited out for the reward is signaled (by pitch of tone for mice and rats, by a number or a “download” bar for humans) and is random (uniformly distributed between 1 s and 30 s) and unknown until entry into the restaurant or gallery.

The computational alignment across species within these tasks offers a platform for translational research aimed at understanding how underlying decision processes change in psychiatric disorders. Recent studies have found that humans with various psychiatric disorders behave differently on these tasks^18,27–29^. Understanding the decision-making processes that underlie the observed behavior on these tasks can provide insight as to how these computational processes change in psychiatric disorders.

**Fig. 1:**
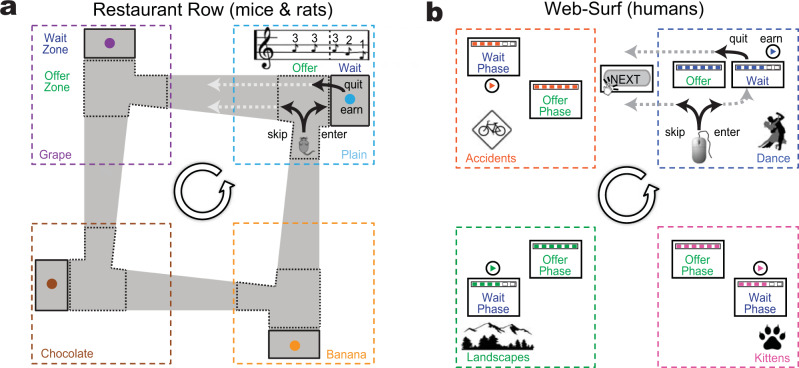
The Restaurant Row and Web-Surf tasks. **a** The Restaurant Row task for mice and rats. As the animal enters the offer zone, the delay is indicated by a tone, which only starts counting down on entering the wait zone. Animals encounter restaurants serially by proceeding around the cycle counterclockwise. **b** The Web-Surf task for humans. An offer is provided to the human as a download bar with a set delay, but the download does not start to countdown until they select “enter” to enter the wait zone. Humans encounter the video galleries serially by clicking through a sequence of buttons. Note the topological analogies. Figure reprinted from^19^ with permission of the publisher.

Multiple variants of these tasks exist, including versions in which there are separate offer and wait stages^16,18,19,23,26^. In these versions, rodents were given two zones at each restaurant, while humans were given two stages to each gallery (Fig. 1a, b). For simplicity, we will refer to these as the “offer zone” and “wait zone”, with the understanding that for humans the two components are separated logically rather than physically. On entering the offer zone, the subject was informed of the delay, but the countdown did not decrease while subjects remained in the offer zone. Subjects could skip as before, leaving the offer zone for the next restaurant/gallery or they could accept by entering the wait zone, at which point the delay began to countdown. Importantly, subjects in this variant could quit the wait zone even though they had accepted the offer by entering into the wait zone, at which point the countdown stopped and the offer was rescinded.

Analyzing behavior on this two-zone variant, we found that the decision to *quit* depended not only on the time remaining in the countdown but also on the time that had already been spent in the wait zone on a given trial—that the longer the subject had spent within the wait zone, the less willing they were to quit, even for identical future conditions in which the time remaining to receive reward was the same^19,26^. Time already spent waiting is the definition of sunk costs^1,2,4^.

**Fig. 2:**
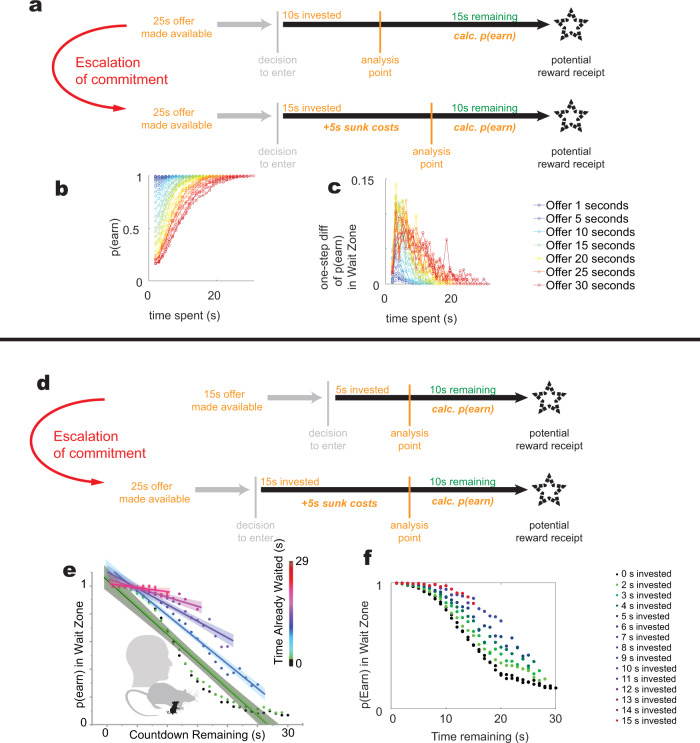
Escalations of commitment on foraging tasks. Once a subject is waiting out a delay, two escalations of commitment can be measured on these tasks. At a given point within the countdown in the wait zone, we ask what is the likelihood that the subject will wait out the delay to receive the reward. This is *p*(Earn). **a** As an agent approaches the reward, it becomes less likely to quit (*p*(Earn) increases). **b** The probability of waiting out the delay (*p*(Earn)) increases with the time spent. **c** This increase is not a simple exponential decrease of quitting, but increases non-linearly with time-spent. **d** Comparing the same future (10 s remaining before receiving the same reward) with different times already invested can also reveal a sensitivity to sunk costs. **e** The evidence for increased *p*(Earn) with time spent (time already waited) originally reported in ref. ^19^. The graph shows the probability of earning, *p*(Earn), as a function of the time remaining in the wait zone. Each colored dot and each line aligned to the colored dots corresponds to the *p*(Earn) given that the subject has waited a certain amount of time in the wait zone already. Notice that the slopes of the lines decrease, indicating that having waited longer, subjects are more likely to wait out the delay, even for a given countdown remaining. Thus, following the logic in **d**, we conclude that the subjects are behaviorally sensitive to sunk costs. **f** The full extent of the data from **e**, showing the continuous “bubble” of increased willingness to wait with time already waited. Models and future comparisons will be plotted in this form, without the linear slope assumption used in^19^. **b**, **c** Replotted from ref. ^30^. **d** Reprinted from ref. ^19^ with permission from the publisher.

Two escalations of commitment are observed on these tasks. First, there is an escalation of commitment as subjects approach the reward^30^ (Fig. 2a). The longer that a subject has waited within the wait zone, the more likely the subject is to wait out the delay (Fig. 2b). While this escalation can be partially explained by the subject getting closer to the reward with passing time, the time course of this escalation (Fig. 2c) is an interaction between the time spent and the time remaining. (Linear model fit, df = 431, F-vs constant model 182, *p* = 10^−76^, effect of time remaining, *p* = 10^−13^, effect of time spent, *p* = 10^−17^, interaction, *p* = 0.0016, adjusted-*R*^2^ relative to constant model = 0.55), implying that it is not simply that the subject is getting closer to the goal. If the escalation were simply due to getting closer to the goal, it should depend solely on the nearness of the goal; it should not depend on the time spent, nor on an interaction between time spent and time remaining.

Second, we can see a difference in commitment between two conditions in which the future path to the goal is identical, but the past is different. Imagine a subject who one time accepts a 15 s offer, waits 5 s, and thus has 10 s more to wait to get to a given goal, and another time accepts a 25 s offer, waits 15 s, and thus has 10 s left to wait for that same goal. Note that the future conditions at these two analysis points are the same—the subject has 10 s to wait for a given reward (the same reward in both conditions). However, when the subject has 10 s to go in the first condition, it has been waiting 5 s (has 5 s of “sunk costs”), while when the subject has 10 s to go in the second condition, it has been waiting 15 s (has 15 s of “sunk costs”). (See Fig. 2d). In the original study^19^, this was measured as the change in linearly fit slopes to the probability of waiting out the delay as the time already waited increased (Fig. 2e), but it is simpler to directly measure the effect through the changes in the probability as an interaction effect between time remaining and time already waited (Fig. 2f). (Linear model fit, df = 461, F vs constant model = 3880, *p* < 10^−100^, effect of time remaining, *p* < 10^−100^, effect of time spent *p* = 10^−42^, interaction, *p* < 10^−100^, adjusted-*R*^2^ relative to constant model = 0.962).

This sensitivity to sunk costs is seen in both the Restaurant Row and Web-Surf tasks and their follow-up variants (such as variants with and without an offer zone^15,19,21^, in the Movie Row task^23^, in the Candy Row task^23^, and in the Known-Delay/Randomized-Delay tasks, in which we manipulated the predictability of the upcoming delay in each restaurant^31^).

## Results

### Sunk cost sensitivity appears primarily in situations where agents are correcting error-made decisions

We do not observe sunk cost sensitivity in all conditions. Nor do we observe sunk cost sensitivity at all times within the wait zone. Nor do we observe sunk cost sensitivity uniformly within the waiting zone. Instead, we find that the sensitivity to sunk costs in the wait zone depends on how economically disadvantageous the decision to accept the offer was in the first place.

The Restaurant Row and Web-Surf tasks (and their variants) are economic foraging tasks, in which subjects are attempting to maximize their consumed rewards within a limited time. Thus, we can identify how worthwhile an offer is to an agent in terms of the delay to the goal (the cost from the limited time budget available) and the (subjective) preference for a given reward. In practice, subjects on these tasks generally reveal a *threshold* for each flavor/gallery, such that they accept delays below that threshold, but reject delays above that threshold. Thresholds can be measured by fitting a logistic regression to the accept vs skip choices, providing both the midpoint (where the accept/skip probability is 0.5) and a slope parameter (measuring the sharpness of the decision logistic). Agents typically showed an individual threshold for each flavor, but these were generally consistent across sessions for rats and mice that ran multiple sessions^15,16,19,22^, and consistent with stated preferences (rankings, ratings) in humans^23,24,26^. Under the assumption that the thresholds measure the individual preferences for each subject, we can thus measure the *value* of an offer, corrected for that individual preference, by subtracting off the threshold. We thus define *value* as the difference between the threshold that the agent has shown for that flavor/gallery and a given offer: **Value** = **threshold** **−** **offered delay**. Thus, offers with delays longer than the threshold for that flavor/gallery have low value and offers with delays shorter than the threshold have high value.

**Fig. 3:**
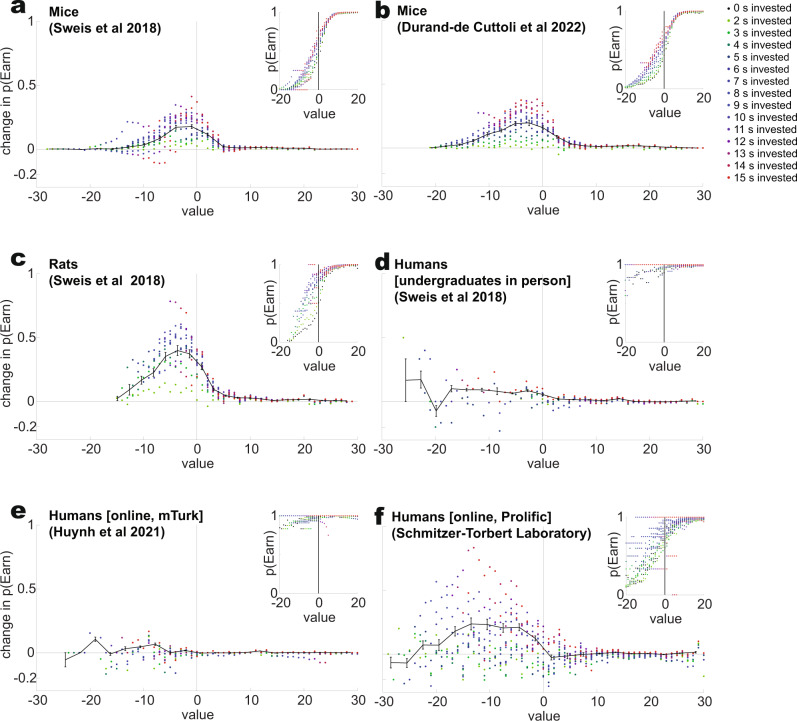
Sensitivity to sunk costs depends on the value remaining. Value is defined as (threshold - offer). Each primary panel shows the increased probability of remaining to earn given the value of a current moment in the countdown. The *x* axis shows the value left in the countdown at the time of quit. The *y* axis shows the change in *p*(Earn) between the time invested and the baseline condition (0 s invested), with time invested marked in color. Insets show the raw probability of remaining to earn at a given value within a countdown. Probability is measured over all subjects. The vertical line marks the transition to where the value crosses zero, where the delay crosses the threshold. Thus, to the right of the line, the remaining time is below the threshold for that restaurant/gallery. Mice, rats, and humans were only sensitive to sunk costs until the value crossed that line. Statistics measured if the change in *p*(Earn) was greater before than after value crossed from negative to positive (Mann–Whitney sign rank test). **a** Mouse data from^16,19^, *p* < 10^−100^ **b** Mouse dataset from^20^, *p* < 10^−100^. **c** Rat dataset from^19^, *p* = 10^−62^. **d** Human dataset from^19^, *p* = 0.27. **e** Human dataset from^23^, *p* = 10^−19^. **f** Online human data from Schmitzer-Torbert laboratory, *p* = 10^−94^.

In Fig. 3, we rearranged the sunk cost sensitivity measure (the increased likelihood of waiting out the delay, the change in *p*(Earn), measured as the difference between *p*(Earn) at a given delay and *p*(Earn) with 0 s invested) by the value that was left at the countdown at the time of quitting. The probability of waiting out the delay to earn is enhanced through sunk costs primarily in the bad deals (deals where the delay remaining was above the typical threshold for that reward site [restaurant or gallery]). (Fig. 3 statistics, Mann–Whitney test, comparing change in *p*(Earn) for values <0 and values >0: 3a (Mice^19^), *p* < 10^−100^; 3b (Mice^20^), *p* = 10^−100^, 3c (Rats^19^), *p* = 10^−62^; 3d (Humans in person^19^), *p* = 0.27; 3e (Humans online^23^), *p* = 10^−19^; 3f (Humans online, NCST dataset), *p* = 10^−94^).

These data suggest that subjects quit out of the wait-zone due to a re-evaluation process, whereby the subject recognizes that they (incorrectly) accepted an offer that was larger than the threshold for that reward. This suggests that one should be able to model this decision with a change-of-mind decision-making model, in which a subject commits to a choice, but then can change its mind and make a different choice if a re-evaluation process occurs and completes before a time-limit^32,33^.

These data suggest that a sensitivity to sunk costs comes through the conflicting decisions of whether to wait out an excessive delay that was accepted incorrectly or to quit. Once the delay during the countdown has crossed the typical threshold, the deal is now seen as reasonable, even if the already spent time is lost, and the sensitivity to sunk costs disappears. We argue that the conflict of whether to rectify the incorrectly accepted deal is driving the sensitivity to sunk costs because the wasted costs lost by rectifying the incorrectly accepted deal are the time already spent (the sunk costs). These hesitation effects are commonly seen in situations of oncoming scarcity threat^34^.

### Sunk cost sensitivity does not solely arise from attrition biases

The strongest evidence for a sensitivity to sunk costs on these tasks is the escalation of commitment seen when comparing the same future outcome, but different times already waited (sunk costs) (Fig. 2d). An important concern, however, is that this effect can only be measured when comparing two different offered delays. Assuming that a subjects’ motivation for each reward can vary over the course of a session, on every encounter with a decision, we can assume that the “willingness to wait” (the motivation for the goal) is drawn from a distribution with some noise. Because subjects are assumed to take deals only when their motivation (their willingness to wait) is above threshold, the distribution of motivations that will drive a subject to accept the longer delay will be different than the distribution of motivations that will drive a subject to accept a shorter delay. Assuming that the agent has variability in its motivation to accept different offers, acceptance of a 25 s deal will require a stronger motivation than acceptance of a 15 s deal.

Ott et al.^35^ argued that this different set of initial motivations would make the set of subjects encountering these two similar analysis points different—the set of subjects encountering the 10 s to go after waiting 15 s would have had to start with a higher subset of the distribution of motivations than the set of subjects encountering the 10 s to go after waiting 5 s. Subjects willing to wait 15 s, but not 16 s or 20 s or 24 s would still have taken the 15 s deal but not the 25 s deal. They point out that this would mean that subjects reaching the 10 s analysis point after waiting 15 s were more likely to have more “willingness to wait” (more motivation) than subjects reaching the 10 s analysis point after only 5 s. Thus, this increase in staying after waiting longer may actually reflect a difference in the willingness to wait for reward as a function of the offered delay to the reward rather than an escalation based on the delay waited. They proposed that the behavioral sensitivity to sunk costs seen in these tasks could be an epiphenomenon consequence of these attrition statistics^35^. They argue that the escalation of commitment shown in Fig. 2e could arise from the distribution of motivations (the willingness to take the deal in the offer zone) in the first place.

Ott et al.^35^ test this theory with a model in which they assume (1) that the subject has a *willingness to wait**** W*** which initially distributes normally around the threshold (**W0** = **W**_**threshold**_ + **N(σ**_**W**_**)**), (2) that the decision to enter an offer occurs when ***W0*** is greater than the threshold for that offer ***T***_**OZ**_, (3) that ***W*** then wanders with a given variance (**σ**_**N**_), and the subject quits if ***W*** ever crosses a quit-threshold in the wait zone ***T***_**WZ**_. Although they do not discuss the importance of the shape of the quit threshold ***T***_**WZ**_ in their model, they assume that the ***T***_**WZ**_ decreases with time so that it starts at the entry threshold ***T***_**WZ**_**(t** = **0) =** ***T***_**OZ**_ and reaches 0 at the time of reward ***T***_**WZ**_**(t** = ***T***_**OZ**_**)** = **0**. Without loss of generality, we can assume a given threshold and examine the sunk costs seen at a single restaurant/gallery. (Ott et al.^35^ assume **W**_**threshold**_ = 18 s, which provides a threshold of 18 s for that modeled restaurant).

**Fig. 4:**
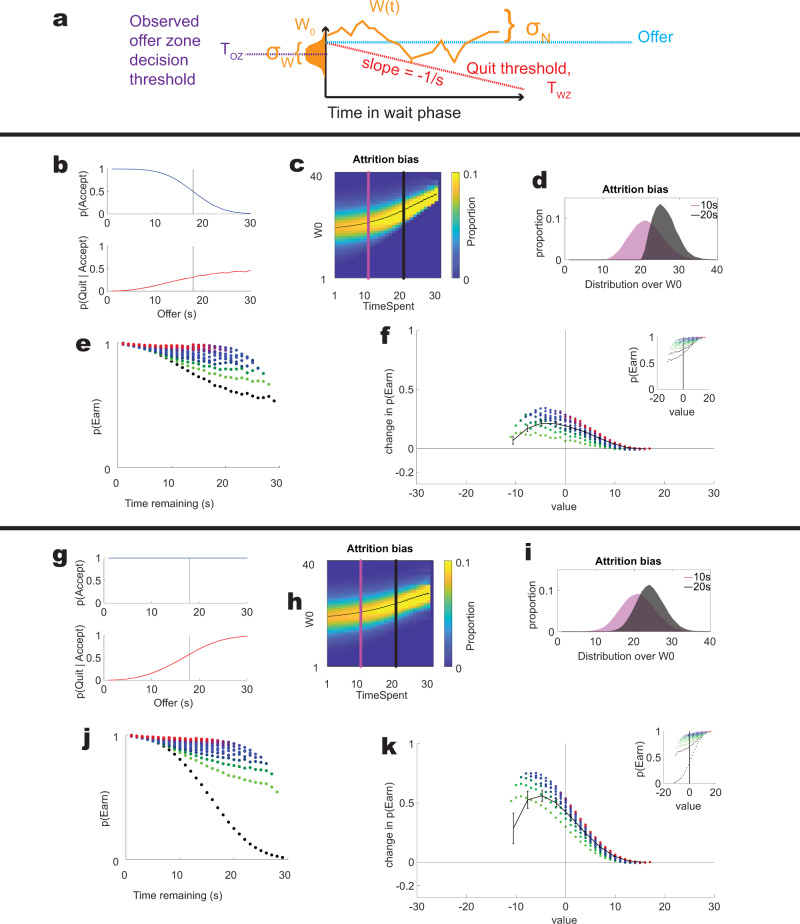
The basic structure of the Ott et al.^35^ model. **a** The basic layout of their model, separating the original enter decision and the leaving decision as dependent on willingness to wait parameters. **b** In the original model, agents probabilistically accept offers based on the initial ***W0*** > *offer* and quit when the wandering **W** crosses the quit threshold **T**_**WZ**_. **c** This produces an attrition bias in their model, in that with longer times-spent in the wait zone, lower initial motivations are no longer part of the distribution. In the panel, each column sums to 1 and is thus a probability distribution over the remaining initial motivations (proportion of the distribution indicated by color as specified in the colorbar). **d** Two example distributions drawn from the set in **c**. Note that the distribution remaining at 20 s time-spent is shifted relative to that available after 10 s time-spent. **e** The model also shows a sensitivity to sunk costs, qualitatively comparable to those seen in Fig. 2f. **f** The model also shows that sunk costs are increased before the value crosses from negative to positive. **g**–**k** This effect is not due to selection bias, but rather to other factors in the model. In a version where the agent accepts all deals (**g**), the attrition bias is reduced (**h**, **i**), but the sensitivity to sunk costs are still as strong (**j**), and the model still shows an increased change in *p*(Earn) for negative over positive values (**k**). Results shown are from our simulations.

We concur that their model does show a sensitivity to sunk costs (see Fig. 4). However, two important questions remain: First, *what are the factors within their model that create this sensitivity to sunk costs?* Second, *how well does their model describe the data we have observed in mice, rats, and humans?*

We start by noting that the Ott et al.^35^ model is a standard drift-diffusion change-of-mind model with some additional parameters (Fig. 4a). Change-of-mind models assume that decision variables continue to drift after a decision is made and that change-of-mind occurs when the decision variable crosses a threshold^32,33^. Their model starts after the decision has been made, and thus assumes that the motivation must have been larger than the threshold. It then drifts until it crosses the quit threshold or the agent receives reward.

Their model has two interesting features: (1) it has an expanding boundary (threshold), such that the decision variable has to move farther away from its starting point in order to reach the boundary the longer the agent has been in the wait zone, and (2) there is only a lower, but not an upper boundary, allowing the decision variable to drift farther away from the boundary the longer the agent has been in the wait zone. Both of these components make the decision variable explicitly and directly sensitive to sunk costs.

These two components (the quit threshold ***T***_**WZ**_ expanding away from the decision variable and the wandering ***W*** component of the drift diffusion model) contain information about the time spent within the wait zone. Because decisions are made based on these processes, the model is less likely to quit the longer the agent has waited (Fig. 4e, f). Thus, the model proposed by Ott et al.^35^ shows a sensitivity to sunk costs because it makes decisions based on parameters that accrue with sunk costs. Interestingly, in the model, these two components do not start to accrue until the agent enters the wait zone, consistent with the observations that sunk costs do not start to accrue until the subject commits to the decision by entering the wait zone^19,20,23,26^.

Ott et al.^35^ claim that the sensitivity to sunk costs arises from the attrition bias that occurs in their model. As can be seen in Fig. 4b–d, there is a selection bias inherent in their model—accepting an offer with a high threshold implies that ***W0*** was selected from the higher part of the distribution **W**_**threshold**_ + **N(σ**_**W**_**)**. As noted above, this implies an attrition in the distribution of ***W0*** with time spent because the shorter times-spent (which can be accepted with lower **W0**) will fall away as the subject crosses that delay. That is, a subject can only reach a 15 s delay for offers of longer than 15 s—shorter delay offers would have provided reward already. To determine whether these effects arose directly from the selection process in taking deals (that the agent only enters the wait zone if ***W0*** was greater than the offer), we tested a version of the model in which the agent takes every choice (Fig. 4g–k). Note that these agents are still quitting if ***W*** < ***T***_***WZ***_, but that includes them in the measurement of sunk costs (in comparison to the agents who do not enter the wait zone at all, and thus are not included in that measurement). This change reduces, but does not eliminate the attrition bias (Fig. 4i); however, this change, if anything, increases the observed sunk costs (Fig. 4j, k), suggesting that there must be other factors in their model producing sunk cost sensitivity beyond the attrition bias.

**Fig. 5:**
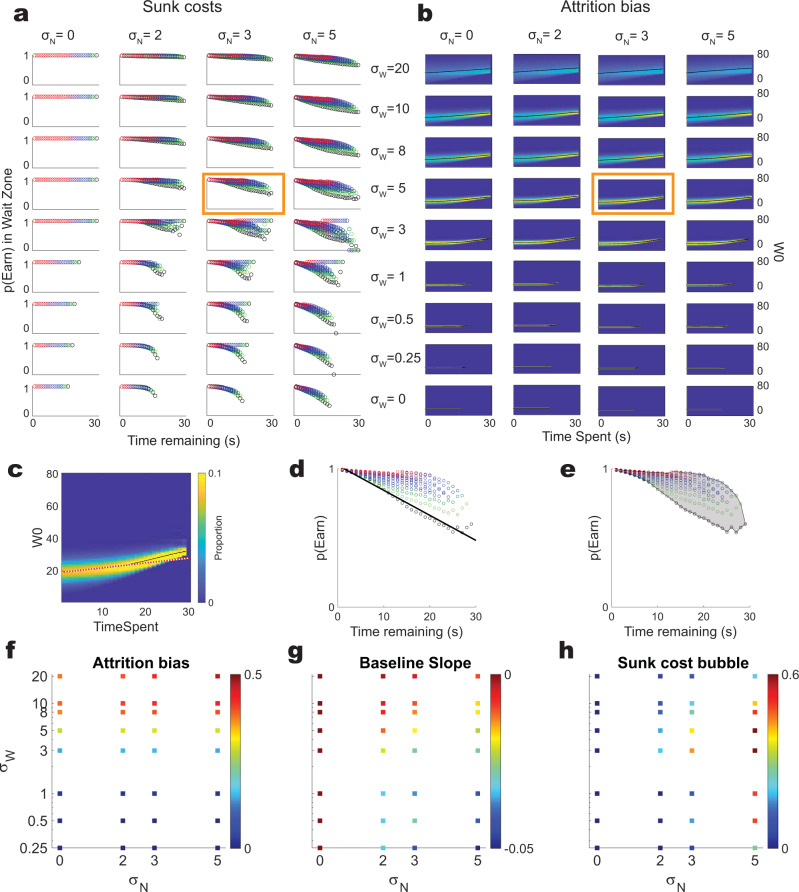
A parameter exploration of the Ott et al.^35^ model. The two key parameters of the model are **σ**_**W**_, the variability in the initial motivation **W**_**0**_, and **σ**_**N**_, the variability in how that motivation changes with time waited in the wait zone. We tested these parameters over a large parameter range (**σ**_**W**_ ∊ {0, 0.25, 0.5, 1, 3, 5, 8, 10, 20} x **σ**_**N**_ ∊ {0, 2, 3, 5}). **a** The sunk cost “bubbles” for each parameter pair. Note that some parameter pairs show more sunk cost sensitivity than others. Colors as per Fig. 2f. **b** The attrition bias histograms for each parameter pair. Note that some parameter pairs show more attrition bias than others. **c**–**e** Measurements used to quantify these simulations. The orange box in a and b indicate <**σ**_**W**_ = 5, **σ**_**N**_ = 3>, which are the parameters used in^35^ and shown in Fig. 4. **c** The attrition bias measured as a linear function of all of the samples providing data to the histogram. Attrition bias slope was measured from the actual points included in the histogram rather than the post-calculated histogram. **d** The “baseline slope” measures how much more likely the agent will wait out the delay as a function of time remaining with 0 s invested. **e** The sunk cost “bubble”, measures sunk cost sensitivity as the summed increased likelihood of waiting as a function of time waited. **f**–**h** How these measures change across the **σ**_**W**_ x **σ**_**N**_ parameter space. **f** Attrition bias is primarily related to **σ**_**W**_. **g** The baseline slope is related to both **σ**_**W**_ and **σ**_**N**_. **h** The sunk cost bubble is largest at high **σ**_**N**_ and a mid-range of **σ**_**W**_. Results shown are from our simulations.

The Ott et al. model contains two important parameters: **σ**_**W**_, the variability in the initial willingness-to-wait that controls the spread of the **W0** Gaussian, and **σ**_**N**_, the variability that controls the drift in **W** as the agent waits in the wait zone. The simulations reported in^35^ used one pair of parameters (**σ**_**W**_ = 5, **σ**_**N**_ = 3), which produce results that are reminiscent of the data shown in Fig. 2e. A thorough exploration of the parameter space finds that the sensitivity to sunk costs are dependent on these two parameters (Fig. 5a), as is the attrition bias (Fig. 5b). However, the attrition bias and the sunk cost sensitivity in this model are unrelated.

We can measure three key parameters of the decisions made in one of these simulations: the “sunk cost bubble”, the “baseline slope”, and the “attrition bias”. We measure the attrition bias as the linear fit of the available initial willingness to wait **W0** as a function of time waited (Fig. 5c). We measure the baseline slope as the linear slope of the 0 s condition (Fig. 5d). And we measure the sunk cost bubble as the summed area comparing the increased likelihood of waiting out the delay relative to the 0s-waited condition (Fig. 5e).

As shown in Fig. 5, attrition bias is most strongly related to **σ**_**W**_ (Fig. 5f, linear model: df = 33, related to **σ**_**W**_: *F* = 92, *p* = 10^−11^, related to **σ**_**N**_: F = 0.2, *p* = 0.65), while the baseline slope depends on both **σ**_**N**_ and **σ**_**W**_ (Fig. 5g, linear mode, df = 33: related to **σ**_**W**_: *F* = 34, *p* = 10^−6^, related to **σ**_**N**_: *F* = 65, *p* = 10^−9^). Sunk cost sensitivity, however, is more related to **σ**_**N**_ and the relationship to **σ**_**W**_ is non-linear, because it is largest at large **σ**_**N**_ and mid-ranges of **σ**_**W**_ and smaller at high and low **σ**_**W**_ (Fig. 5h, linear model, df = 33: related to **σ**_**W**_: F = 0.01, *p* = 0.9, related to **σ**_**N**_: *F* = 56, *p* = 10^−8^). Thus the highest sunk cost sensitivity appears at <**σ**_**W**_ = 3, **σ**_**N**_ = 5> Most importantly, however, the sunk cost sensitivity is not linearly related to the attrition bias (linear model: df = 34, *F* vs constant model = 1.01, *p* = 0.32, adjusted-*R*^2^ relative to constant model = 0.0004). Thus, attrition bias is neither necessary nor sufficient to create a sensitivity to sunk costs within their model.

So if the attrition bias of the willingness to wait parameter is not causing the sensitivity to sunk costs in this model, what is? As noted above, in the model, the **quit threshold** ***T***_**WZ**_ decreases from being equal to the offer on entry into the wait zone to 0 at the time of reward, providing an expanding boundary to the drift-diffusion change-of-mind model. The quit threshold decreases at a constant rate of 1/s. Note that agents enter the wait zone with a willingness to wait, **W0**, that is going to be above the offer (because of the process by which the model accepts an offer). As the agent spends time in the wait zone, ***T***_***WZ***_ decreases so that it will be 0 when the countdown stops. This means that ***T***_***WZ***_ moves away from ***W0*** at a rate of 1/s as the agent waits in the wait zone, making the agent less likely to quit the longer it spends in the wait zone. (This is the literal definition of a “sensitivity to sunk costs”).

**Fig. 6:**
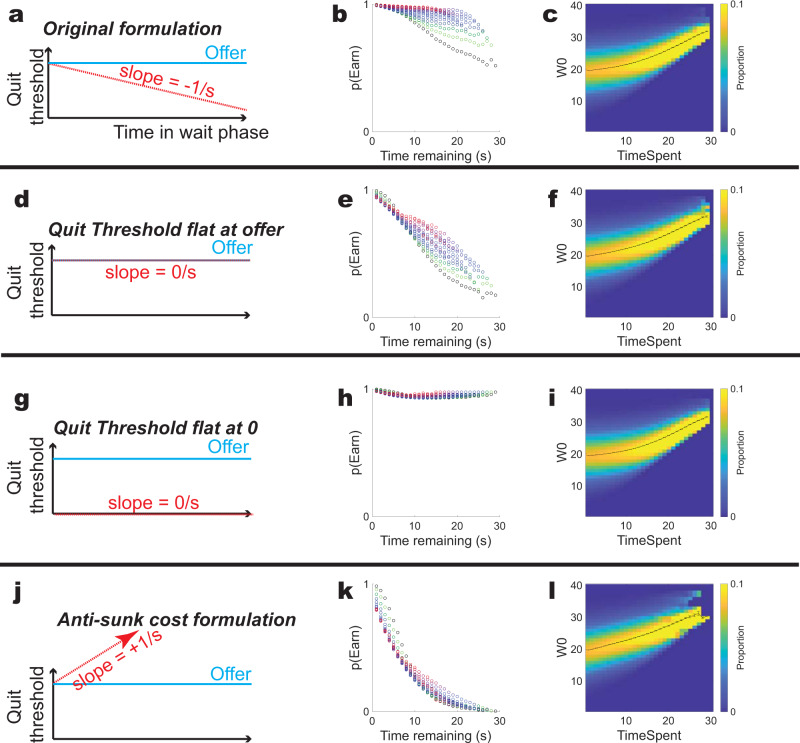
Effect of changing the slope of the quit threshold T_WZ_ in the Ott et al. model. **a** The original formulation. **b** The sunk cost sensitivity seen in the original formulation. **c** The attrition bias seen in the original formulation. **d**–**f** Effect of removing the slope of **T**_**WZ**_, while starting **T**_**WZ**_ at the offer, thus making the quit threshold equal the offer for the entire time in the wait zone. **g**–**i** Effect of removing the slope of **T**_**WZ**_, while starting **T**_**WZ**_ at 0, thus setting the quit threshold to 0 for the entire time in the wait zone. Note that because **W** can wander to a number less than 0, it is still possible for the agent to quit. **j**–**l** An anti-sunk-cost formulation in which the **T**_**WZ**_ bound increases at a rate of 1/s, providing a collapsing bound to the drift-diffusion model. The sunk cost bubble is inverted with lower *p*(Earn) the longer the agent waits in the zone. Note that even the anti-sunk-cost formulation shows attrition biases. Time-invested colors in **b**, **e**, **h**, **k** as in Fig. 2f. Results shown are from our simulations.

Importantly, this means that the changing threshold contains information about both the time remaining and the time spent (Fig. 6a–c). When we remove this rate of change (removing the information about time spent as we remove the information about time remaining), we find that this decreases the sensitivity to sunk costs. If we set the quit threshold to the offer, we decrease the sensitivity to sunk costs (Fig. 6d–f). If we set the quit threshold to 0, the agent never quits (Fig. 6g–i). A large part of the reason that their model is sensitive to sunk costs is that such a sensitivity is directly built into the model with the changing quit threshold. In order to produce an agent that quits but does not show sunk costs, we need to include an anti-sunk cost formulation, wherein the quit threshold increases with time spent rather than decreases, making it easier to quit the longer the agent is in the wait zone (Fig. 6j–l), suggesting that there is an additional factor critical to the presence of sunk costs in the model.

**Fig. 7:**
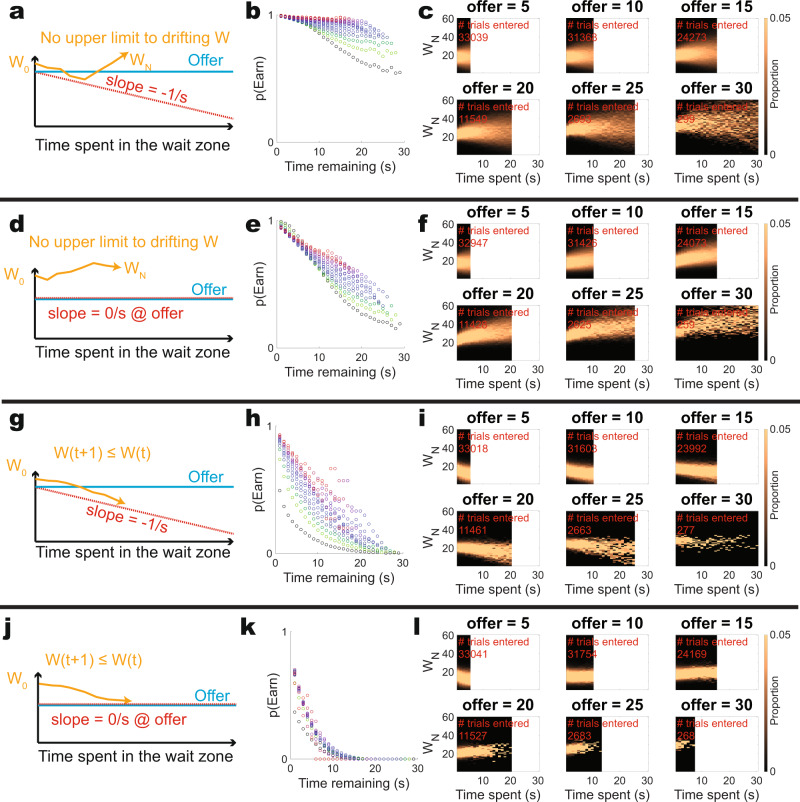
Limiting the drift of the willingness to wait. **a** The original formulation limits the drift of the willingness to wait at the lower bound by **T**_**WZ**_, decreasing by 1/s. **b** This produces sunk cost sensitivity. **c** The distribution of **W** over time as a function of offer. Note the increasing variance with time, containing information about the time spent. **d**–**f** The formulation used in Fig. 6d. The distribution of **W** over time as a function of offer continues to show the increasing variance with time, containing information about the time spent. Samples drift away from the lower bound, making it more difficult to quit with time (i.e., making the decision sensitive to sunk costs). **g**–**i** A formulation with the original lower bound, but a new upper bound, restricting the change in **W** to always decrease. If **W**(t) was slated to rise above **W**(t-1), it was set to **W**(t-1). Note that there is still information about sunk costs due to the time information in the descending lower bound slope. **j**–**l** If we include both flat upper and lower bounds, the sensitivity to sunk costs disappears. Time-invested colors in **b**, **e**, **h**, **k** as in Fig. 2f. Results shown are from our simulations.

The other factor that is critical to the presence of sensitivity to sunk costs in this model is that the model has only a lower boundary but no corresponding upper boundary. This means that as time passes, the drifting willingness to wait is more likely to drift away from the threshold (Fig. 7). Thus, the variance of the term ***W*** contains information about the time spent. Because the decision process in their model is made sensitive to that information through the use of a lower but not an upper bound, their agent is less likely to quit the longer it spends in the wait zone. (Again, this is the literal definition of a “sensitivity to sunk costs”). To test the extent to which this variability drives the sensitivity to sunk costs in this model, we examined limiting the maximum drift of the willingness to wait **W** over the time waiting, that is, removing that sunk cost information from the decision process. Figure 7 shows that the sensitivity to sunk costs arises from the distribution of the decision variable **W** over time, which has been made directly sensitive to sunk costs in the model due to the expanding lower bound (the decreasing slope of **T**_**WZ**_) and the increasing variance of **W** with time due to the lack of an upper bound. (Original model, Fig. 7a–c, limiting only the quit threshold, Fig. 7d–f, limiting the upper bound, Fig. 7g–i, limiting both, Fig. 7j–l).

Figures 6 and 7 show that the sensitivity to sunk costs in the model is due to the decision processes that were made sensitive to the time spent waiting (the sunk costs). The decision process is sensitive to the expanding bounds, which make it harder to quit the longer the agent is in the wait zone—a lower bound that expands away from the offer with a rate of −1/s controlled by **T**_**WZ**_, and an upper bound that is driven by the ability of the ***W*** to walk away from the initial willingness-to-wait ***W0*** that is controlled by **σ**_**N**_.

### Comparing the model to data

The preceding sections illustrate that sunk cost sensitivity within the model proposed by Ott et al. is not related to attrition bias but instead depends on decision variables/parameters that represent the time waited (the costs that have been sunk). Nevertheless, their model can serve as a starting approach to examine decision processes underlying sunk cost in mice, rats, and humans. To address this possibility, we assessed how well the parameterizations in their model can fit the data from individual subjects.

### Individual variability in the sensitivity to sunk costs

Importantly, we also find that there is a lot of individual variability in each subject’s sensitivity to sunk costs, with some mice, rats, and humans being particularly sensitive, and others not^16,19,20^. These differences likely arise from variations in cognitive and behavioral factors, such as attention, predictability, baseline motivation (such as hunger), and other factors. Kazinka et al.^26^ tested humans online under two conditions, one in which they were asked to attend to a button that could change color subtly, and another without such an attention-check. When humans were distracted from the delay countdown, we found that the sensitivity to sunk costs disappeared, but that it remained intact in the version without the attention-check^26^. In rodents, we found differences in the sensitivity to sunk costs as a function of environmental richness (where the distribution of delays was shifted lower so more food could be gathered more quickly or shifted higher requiring more time to gather food)^16,36^. Rodents also exhibit differences in sunk cost sensitivity as a function of the predictability of the upcoming delay—delays that could be predicted produced less sensitivity to sunk costs than delays that were only revealed on entry into the restaurant^31^.

As noted above, the Ott et al.^35^ model provides two key parameters (**σ**_**N**_ and **σ**_**W**_) that produce different sunk cost sensitivities (Fig. 5). While it is clear that the parameters chosen by Ott et al.^35^ [**σ**_**N**_ = 3 and **σ**_**W**_ = 5] would not produce this variation, it is possible that different parameter configurations could capture the variability in sunk cost sensitivity seen across subjects.

**Fig. 8:**
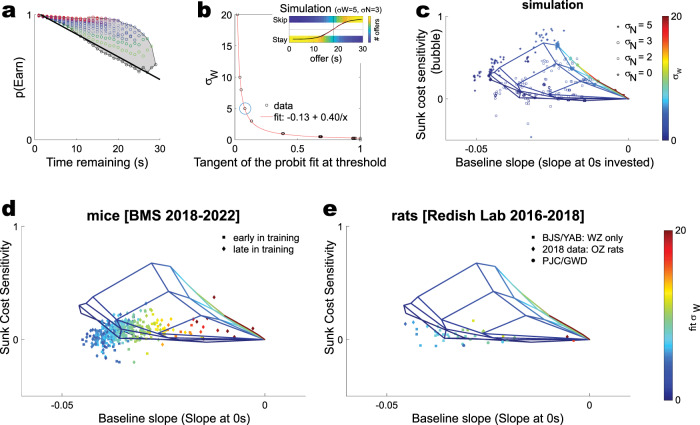
Individual variability. The basic model proposed in^35^ has two parameters **σ**_**W**_ (which controls the offer zone accept/skip decision) and **σ**_**N**_ (which controls the drift rate within the wait zone). **a** As shown in Fig. 5, each of these parameters produces a sunk cost sensitivity, measurable as the area of the increased likelihood of earning as a function of the time invested (the “sunk cost bubble”), and a specific slope of the likelihood of quitting at 0 (“baseline slope”). **b** Although we know of no way to measure **σ**_**N**_ from the behavioral data directly, **σ**_**W**_ is directly related to the slope of the psychophysics curve of accepting an offer. Inset panel shows the original model (<**σ**_**W**_ = 5, **σ**_**N**_ = 3 > ), the histogram of offers for which the agent stayed, offers for which it skipped, the fit probit model (in black), and the slope at threshold (in red). **c** Thus, for each simulation we can measure the “sunk cost bubble”, the “baseline slope”, and **σ**_**W**_. We ran 20 simulations at each part of the parameter space from Fig. 5. Symbols for each simulation are shown. The mesh grid indicates the average for each parameter pair. Color on the mesh is interpolated from **σ**_**W**_. **d** Mice from ref. ^19,20^. Each mouse provides one symbol from early in training and one symbol from late in training, placed at the measured baseline slope and sunk cost sensitivity. Color is the fit **σ**_**W**_, given the slope at threshold. *n*(mice) = 208. **e** Rats from^19,21^ (*n* = 21 rats with wait zone only, 10 rats with both wait and offer zones) and new data from the Redish lab (*n* = 15 rats).

Although we know of no behavioral way to directly measure the **σ**_**N**_ parameter, the **σ**_**W**_ parameter is related to the willingness to take economically inconsistent offers above and below the observed threshold, which appears as the slope of the psychophysics decision curve of accept vs skip decisions made in the offer zone. That is, we can consider a subject’s decisions to accept or reject an offer as a standard decision process which shows a typical psychophysics curve with a slope β. The flatter the slope, the more likely the subject will pick a choice inconsistent with its personal threshold. Because the decision in the Ott et al. model is based on whether **W0** > threshold or not and **W0** is drawn from a distribution with variance **σ**_**W**_, the slope will be directly related to that variance **σ**_**W**_. To confirm this, we ran the simulation with 10 samples each of varying **σ**_**W**_ parameters and measured the tangent at the 50% threshold of a fit probit model. We found a remarkably good relationship (Fig. 8b), such that **σ**_**W**_ ≈ **−0.13** + **0.40**/**x**, where **x** is the tangent of the probit model at the threshold (i.e., the point where the probability of skipping versus accepting an offer as a function of the delay offered is 50%). Importantly, this tangent can be directly measured from data to derive an estimate of **σ**_**W**_ for each subject.

Humans only experience one session each, and thus we do not have enough data to fit sunk cost sensitivity for each individual subject. (The human data sets measure sunk cost sensitivity over the whole population^19,23,26^. However, the rats and mice each run the Restaurant Row task for many days^16–18,20,21,30^, and thus, we can measure the three key parameters for each individual subject. We can measure (1) the sunk cost bubble and (2) the baseline slope (Fig. 8a) from the choices made by the subject. (3) We can estimate **σ**_**W**_ from the tangent at the probit fit of the subject’s threshold (Fig. 8b). Thus, each subject can be placed on a three-dimensional plot of baseline slope, the sensitivity to sunk costs, and the fit **σ**_**W**_.

To compare the model’s ability to capture the individual variability, we first ran 20 simulations of each parameter instantiation of the model (**σ**_**W**_ ranging over {0, 0.25, 0.5, 1, 3, 5, 8, 10, 20}, and **σ**_**N**_ ranging over {0, 2, 3, 5}, as shown in Fig. 5). We then measured the average baseline slope and sunk cost sensitivity for each simulation. We constructed a mesh grid of the mean at each <**σ**_**W**_,**σ**_**N**_ > pair, color coded by **σ**_**W**_ (Fig. 8c). Repeating this grid for the mice (Fig. 8d) and the rats (Fig. 8e) shows that the actual observed data does not track the grid very well.

To compare these statistically, we found the closest mean point on the simulation matrix mesh for the <**σ**_**W**_,**σ**_**N**_ > with the fit **σ**_**W**_ for the individual, and then measured the distance between that point and the individual’s baseline slope and the distance between that point and the individual’s sunk cost sensitivity. If these differences are due to noise, they should center at 0. We thus measured whether those differences were centered at 0 with a Mann-Whitney sign rank test. Every dataset was significantly different in either the baseline slope or the sunk cost sensitivity or both. (Fig. 8d: *n* = 208 mice early in training^19^ *p*(sunk cost sensitivity) = 0.12, *p*(baseline slope) = 10^−35^; *n* = 208 mice late in training^19^ *p*(sunk cost sensitivity) = 0.17, *p*(baseline slope) = 10^−35^; Fig. 8e: *n* = 21 rats without an offer zone^19^ *p*(sunk cost sensitivity) = 0.5, *p*(baseline slope) = 10^−5^; *n* = 10 rats with both zones^19^ *p*(sunk cost sensitivity) = 0.04, *p*(baseline slope) = 0.002; *n* = 15 rats with both zones (new RedishLab rat data) *p*(sunk cost sensitivity) = 0.0001, *p*(baseline slope) = 0.00001). As can be seen in these statistics, the model can only match the sunk cost sensitivity by showing completely incompatible baseline slopes.

### Sunk cost sensitivity and the offer zone

The most important result that came from the introduction of the offer zone variant^19^ is that we did not see a sensitivity to sunk costs in the offer zone. Subjects spent time deciding in the offer zone even though time spent in the offer zone counted against the total economic time budget that the subjects could use to forage for food. However, time spent in the offer zone did not show a sensitivity to sunk costs; the calculation of time spent (the cost that had been sunk) did not start to accrue until the agent entered the wait zone. This result has been replicated in online samples^23,26^ and subsequent experiments^20^. In general, time spent in other portions of the task did not influence the probability of quitting after accepting an offer, implying that time spent in the wait zone carries a unique sensitivity to sunk costs^30^.

As we have argued elsewhere^16,19,26^, the most parsimonious explanation is that behavior in the offer and wait zones arise from different decision processes, consistent with both multiple-decision theories^37,38^ and commitment hypotheses^39,40^. Importantly, it is not rational to spend any time in the offer zone, as any time spent making decisions in the offer zone could be more productively spent being made in the wait zone, while the delay is counting down. However, all three species spend more time than necessary in the offer zone, and mice and rats, at least, increase their time spent in the offer zone with experience^16,19^. One possible explanation for this is that it is difficult to quit out of the wait zone due to the accumulation of sunk costs^16,17,19,36^.

As shown in Figs. 4–7, the reason that the model laid out in ref. ^35^ shows an escalation of commitment with time spent is not due to the theoretical arguments made by Ott et al. therein (that sunk cost sensitivity seen on the Restaurant Row and WebSurf tasks is due to a statistical attrition of initial motivation) but rather to decision processes built into the model that are explicitly dependent on time spent in the wait zone (i.e., sunk cost). Nevertheless, even though the model proposed in^35^ is incompatible with the theory proposed in^35^, the model itself can provide an interesting explanation for the difference between the offer and wait zones because it includes change-of-mind decision processes^32,33^ that are explicitly sensitive to time spent in the wait zone, but not the offer zone. In that model, the offer zone decision is made based on the initial willingness-to-wait ***W0***, which starts to drift once the agent enters the wait zone. Similarly, the quit threshold ***T***_***WZ***_ only starts decreasing in the wait zone. Thus, the decision process is sensitive to the time spent in the wait zone, but not the offer zone.

### Sensitivity to sunk costs is delayed in variants without an offer zone

In a recent variant without an offer zone, we directly manipulated the upcoming uncertainty of the future outcomes^31^. In two parallel tasks, rats approached a series of reward sites which either had a known delay (different across the four reward sites, but constant within each reward site for a given day, changing from day to day, thus predictable after experiencing one lap around the maze) or had a random delay to reward (1s–30s random on each entry, thus unpredictable). In the first task (*Known-Delay [KD]*), rats knew what the upcoming delay would be and showed behaviors indicating that they had already made their decision before entering the reward zone—they maintained a fast speed on approach to offers they skipped. In the second task (*Random-Delay [RD]*), rats slowed down on every entry into an offer and then only sped up to leave after a few seconds of consideration. Furthermore, decisions in the KD task were more self-consistent, that is, rats were more likely to either choose to stay or skip for a given delay on the KD task. We found that sunk cost sensitivity was decreased in the KD task, but were still present. Importantly, however, we also found that sunk costs did not start accruing in the RD task until after the decision-time (~5 s), while sunk costs started accruing in the KD task immediately.

That subjects make a separate decision before committing to a choice and then show a sensitivity to sunk costs only after commitment^16,19,26,39,40^ suggests that one should see this difference, even without an explicit offer zone. And, in fact, that is what has been found. The early versions of the Restaurant Row and Web-Surf tasks did not have a separate offer zone, and only included a wait zone^15,21,24^. A reanalysis of these data found that sunk costs did not start accruing for several seconds^19^. We hypothesized that animals were spending the first few seconds making an *accept/skip* decision as if they were in an offer zone rather than a *quit/remain* decision. We hypothesized that they then covertly transitioned to a *quit/remain* decision and began building sunk costs. We found robust sunk cost sensitivity in these animals, but only after a delay, consistent with typical decision times made in variants with an explicit [separate] offer zone.

### Delayed accrual provides an opportunity to test the reset hypothesis

**Fig. 9:**
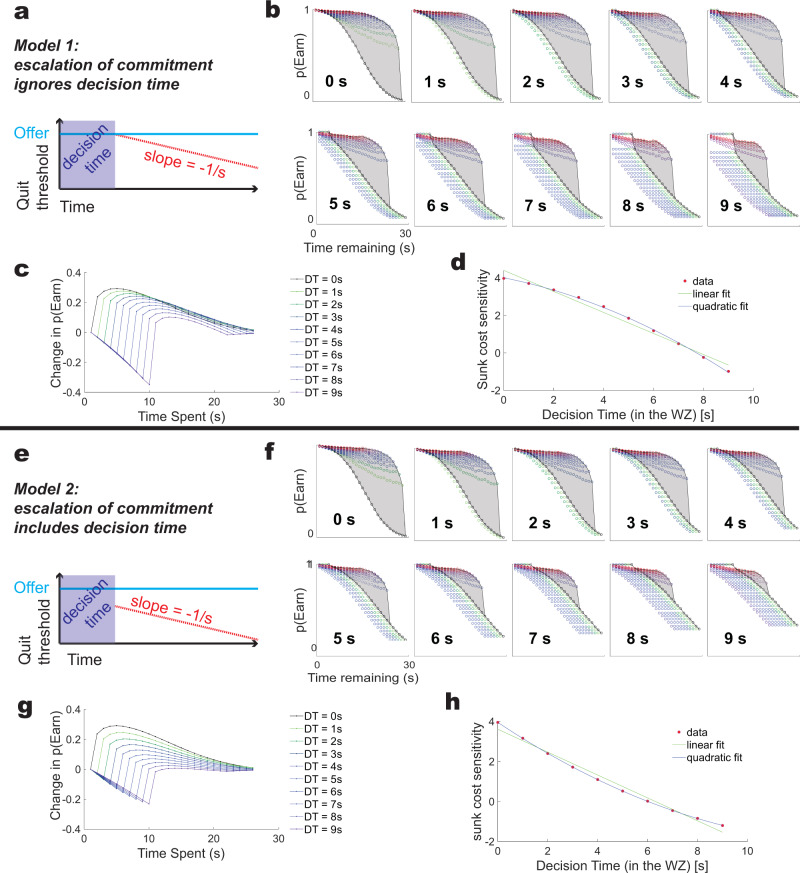
A delay to accrual allows us to test the reset hypothesis. In variants without an offer zone, and in some subjects with limited experience with a separate offer zone, subjects do not quit for a short period of time in the wait zone (a few seconds). This short non-quit time in these subjects can be theorized to be a separate decision process^15,16,19,36^. Modeling this as an inability to quit for a few seconds opens up two models: *Does the escalation of commitment take that decision time into account or not?* **a** Model in which sunk cost accrual starts only when the decision time (DT) is over. **b** The “sunk cost bubble” changes as a function of the delay before the agent can start quitting. Each panel shows a decision time of x seconds. **c** Comparing the total change in *p*(Earn) [the size of the sensitivity to sunk costs] as a function of time spent and a given decision time shows that these sensitivities are dependent on both time spent (time invested, costs sunk) and the decision time. **d** For each given decision time, we summed the total sunk cost sensitivity. This shows a significantly convex curved fit (linear fit adjusted-*R*^2^ = 0.98, quadratic fit adjusted-*R*^2^ = 0.99). **e** Model in which sunk cost accrual includes the decision time. **f**–**h** This produces a significantly concave curved fit (linear fit adjusted-*R*^2^ = 0.98, quadratic fit adjusted-*R*^2^ = 1.00).

This delayed latency provides an opportunity to address a question raised by Ott et al. in their paper — when the subject finally makes the decision to stay in these single-zone variants, they have already been in the zone for several seconds. So we can ask whether the subsequent quit or remain decisions take into account the initial decision time. (Remember that we do not see sunk cost accrual during similar decisions made in the separate offer zone). We can test this by comparing two variants of the Ott et al. model: one in which ***T***_***WZ***_ starts descending only after completion of the self-imposed (hidden) decision-time (Fig. 9a) and one in which ***T***_***WZ***_ has already been descending for those several seconds (Fig. 9e). In other words, *does the increased difficulty to quit with time (the escalation of commitment) include that decision time or not?*

We modeled the single-zone construction by having the agent accept all offers (thus ignoring the offer zone, effectively modeling a task variant without an offer zone) and the decision time by simply preventing the agent from quitting for the first few seconds of waiting in the wait zone. Fascinatingly, the two simulations produced fundamentally distinct shapes of sunk cost outcomes, more specifically, the relationship between differences in the latency to accrue sunk costs and maximal sensitivity to sunk costs (Fig. 9d, h). These two models produced a subtle difference in the maximum sunk cost sensitivity levels seen as a function of the time spent deciding. The first model (**T**_**WZ**_ is at offer at the end of the decision time and starts decreasing only after the first-stage decision (to stay) has completed) showed a convex curve as a function of the time spent in the pre-wait-zone/hidden decision time, where particularly short and long decision times were less sensitive to sunk costs than mid-range decision times. (Fig. 9b–d, linear fit adjusted-*R*^2^ = 0.98, quadratic fit adjusted-*R*^2^ = 0.99). In contrast, the second model (**T**_**WZ**_ starts decreasing immediately) showed a concave curve, in which mid-range decision times were less sensitive to sunk costs. (Fig. 9f–h, linear fit adjusted-*R*^2^ = 0.98, quadratic fit adjusted-*R*^2^ = 1.00).

Comparing these simulations to new analyses of data from rats on versions of the task without an offer zone^19,21,22^, and the subsets of mice who took all offers and effectively ignored the offer zone choice stage^16^, we found a fascinating difference between the rats and mice.

**Fig. 10:**
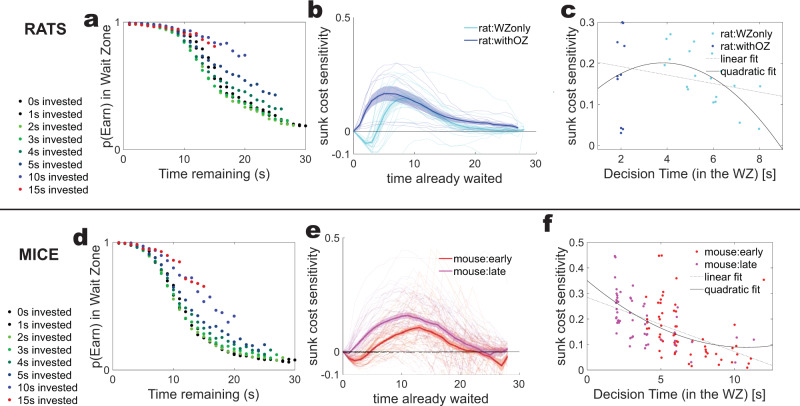
Fitting sunk cost sensitivity by decision time for rats and mice. **a** The sunk cost bubble for one example session from rats without an offer zone. Note that the first few seconds invested are unchanged relative to the 0 s invested condition. Compare Fig. 2f. **b** The sunk cost sensitivity as a function of time spent (time already waited). Note that the rats with the offer zone start accruing sunk costs immediately, but rats without an offer zone do not start accruing sunk costs for a few seconds. Compare Fig. 9c. **c** Fitting this curve by decision time finds a significantly convex curve (*n* = 21 rats without an offer zone and 10 rats with, quadratic adjusted-*R*^2^ = 0.13 > linear adjusted-R^2^ = 0.04), suggesting Model 1 (Fig. 9a) that rats without an offer zone are not including the decision time in the expanding **T**_**WZ**_ boundary. **d** One example mouse session showing that the probability of waiting out the delay given that the mouse has already invested a few seconds is not different from the 0 s invested data. **e** Early in training mice do not accrue sunk costs immediately, but take a few seconds before accruing them. Late in training, mice start accruing more immediately. **f** Fitting this curve by decision time finds a significantly concave curve (*n* = 32 mice, each mouse provides one data point from early training and one from late training, quadratic adjusted-*R*^2^ = 0.31 >linear adjusted-*R*^2^ = 0.29), suggesting Model 2 (Fig. 9d) that mice early in training are including the decision time in the expanding **T**_**WZ**_ boundary. See text for discussion of outlier removal.

The rats showed evidence that they did not start to accrue sunk costs until the decision had been made (Fig. 10a–c). Figure 10b shows that rats without an offer zone (i.e., with only a wait zone) delayed their sunk cost accrual by a few seconds^15,19,31^. We thus measured whether the sunk cost sensitivity was better explained by model 1 (no decay in the quit threshold through the decision time, Fig. 9a) or model 2 (quit threshold decays through decision time, Fig. 9e). There was one data point well apart from the other samples (Latency to Accrual = 29 s, all other data in the range of 4 s to 8 s). With this outlier included, the quadratic fit was not better than the linear fit (linear fit adjusted-*R*^2^ = 0.14, quadratic fit adjusted-*R*^2^ = 0.11); however, with this outlier excluded, the quadratic fit was better and was convex (linear fit adjusted-*R*^2^ = 0.04, quadratic fit adjusted-*R*^2^ = 0.13). Given the single point that was far away from the main distribution, we treat it as an outlier. The rest of the data suggest that rats were treating this “wait-zone-only” version of the task as having a conceptual “offer zone”, and thus that the decision time is not incorporated into the perception of sunk-costs. In other words, the rats did not start to accrue sunk costs until after the decision was made and they had left their self-imposed “offer zone” (consistent with model 1, Fig. 9a). This observation is consistent with previous experiments on rats without an offer zone^15,19,21,31^.

In contrast, the mice showed evidence that sunk costs accrued through the whole time in the wait zone (consistent with the second model that **T**_**WZ**_ starts decreasing immediately) Fig. 10d–f. Figure 10e shows that early in their experience, the mice did not start accruing sunk costs for a few seconds, while late in their training, sunk costs started accruing on entry into the wait zone. This is likely due to the mice learning to use the offer zone to avoid sunk costs in the wait zone^16^. The data shown has outliers removed as per the rat data, below, however, for the mice, both analyses with and without outliers removed showed concave quadratic fits that were better than the linear fits. (Data shown, linear adjusted-*R*^2^ = 0.29, quadratic adjusted-*R*^2^ = 0.31). These results suggest that the mice started accruing their sunk costs immediately and that the escalation of commitment (i.e., increased difficulty to quit) includes the complete time spent in the wait zone (consistent with model 2, Fig. 9e). This has important implications for how the internal value state of the agent has changed during the passage of time while waiting and how this state compares to the perceived value of time left in the countdown registered at the transition point when sunk costs begin to accrue. In other words, when a mouse looked up from the decision time and found itself already in the wait zone for several seconds, it took that total time already waited in its sunk cost calculation, in effect resetting its sunk costs based on the actual time spent.

Importantly, the subset of rats analyzed in Fig. 10 came from an experiment in which they never had an offer zone;^21^ they only had a wait zone. This makes their experience very different from the other subjects analyzed in this paper, including the mice shown in Fig. 10, who had an offer zone available, but had not learned to use it yet, although they did eventually learn to make their decisions in the offer zone^16^.

## Discussion

A sensitivity to sunk costs can be operationally defined as an escalation of commitment with continued time spent in an operation^1,2,4^. An escalation of commitment with time spent waiting for reward (a sensitivity to sunk cost) has been observed in some variants of the Restaurant Row and Web-Surf tasks, particularly in the wait zone but not the offer zone of these tasks^19,23,26^.

Ott et al.^35^ proposed that this observed escalation of commitment may be due to a statistical attrition bias in the initial motivation, such that longer times-spent waiting were correlated with increased initial motivations, thereby resulting in a higher probability of earning reward with more time spent. Our thorough simulations of their model find that while this attrition bias does exist, it is unrelated to the presence or absence of a sensitivity to sunk costs. Although theory in ref. ^35^ proposed that the escalation of commitment seen in these tasks is due to statistical attrition, we show that this is not a viable explanation for the observed phenomenon. Ott et al. also proposed a specific simulation model that shows a sensitivity to sunk costs. Our rigorous analysis of their model finds that sunk cost sensitivity appears in their model due to two phenomena in which decision processes were explicitly made sensitive to time spent (sunk costs): the escalation of commitment arises from the expanding lower boundary provided by the decreasing quit threshold ***T***_***WZ***_ and from the increasing range available to the drifting motivation (willingness to wait, ***W***) over time.

This is not the first model to show how a simple decision process can produce an escalation of commitment and a sensitivity to sunk costs. In fact, several previous animal learning theory models have been put forward that produce similar sunk cost sensitivity effects, including the state-dependent-valuation learning [SDVL]^7,9,41^ and within-trial-contrast [WTC]^42^ models. The SDVL model notes that ongoing energy expenditures imply that food rewards will become more valuable to an agent as time progresses. Thus, reward valuation should depend on the time since the last reward was received. Of course, the SDVL model cannot explain why humans foraging for videos show similar effects, but we can hypothesize a more general “value process” that depends on recency of experience. The WTC model notes that distant rewards are discounted so that approaching rewards are seen as increasing in value, which can provide an increasing contrast between the current state of the animal (changing through SDVL) and the approaching reward^42^.

As noted in ref. ^19^, neither the SDVL nor the WTC models can explain why sunk costs only seem to begin accruing after an initial commitment decision has been made (entering the wait zone of the tasks) and why a parallel sensitivity to time spent is not also present in the offer zone. Moreover, neither model can explain why a lack of attention to the decreasing delay reduces sensitivity to sunk costs^26^, why predictability of upcoming costs reduces the sensitivity to sunk costs^31^, or why the richness of the environment affects the sensitivity to sunk costs^16,20,36^.

The new model proposed in ref. ^35^ may provide an interesting hypothesis. Although it is unclear why the quit threshold expands away from the offer at a rate of 1/s, if the offer zone and wait zone are decided upon by different decision processes^16,18,19,23,26,29,31,43^, this quit threshold that only starts decreasing on entry into the wait zone might provide an explanation for the accrual of sunk costs after commitment. In the versions of the task with separate offer and wait zones, the delay does not begin counting down until entry into the wait zone^16,19,23,26^. Similarly, if a new drift diffusion process begins only with commitment, change of mind behaviors^32,33^ might be sensitive to sunk costs only after that commitment^39,40^, which would be well modeled by the increasing variance of the drift-diffusion process providing more opportunities to have increased motivation with time spent (an escalation of commitment). Applying their model to versions with a single zone (wait zone only, no offer zone) suggests that rats who never experienced an offer zone did not start to accrue sunk costs until making the commitment to stay in the wait zone, effectively treating the initial decision time as a separate offer zone. However, mice that had not yet learned to use the available offer zone showed an accrual of sunk costs that took into account all of the time within that wait zone. Whether this is a species difference (rats vs mice), a difference in the tasks (no offer zone available vs have not yet learned to use the offer zone), or both, remains unknown.

Also present within the data are observations that are incompatible with these simple drift-diffusion models, including the observation that sunk cost sensitivity is stronger when subjects have taken a bad deal^20^ and that some subjects show this escalation of commitment, while others do not^18,26^. We suspect that one potential explanation of the data is the increased motivation provided by Pavlovian associations between the countdown in the wait zone and the value of the outcome. This would predict a decreased sensitivity to sunk costs with reduced attention (consistent with the online human data^26^), as well as a heightened sensitivity to factors that increase that relationship to the reward zone (such as conditioned place preference)^44–47^. This is a form of the endowment effect^48–50^ and is similar to the deliberative vs implementational mindset constructs in the human literature^39,40^. Essentially, this hypothesis suggests that quitting out of the wait zone is a recognition of a mistake and staying is due to an unwillingness to leave that mistake. Because the extent of the mistake depends on the effort spent (more effort spent was a larger mistake), the decision becomes related to the effort already spent and shows a sensitivity to the sunk costs.

It is worth noting that there are situations where an increased motivation from effort already spent can actually be economically useful (see ref. ^4,5,51,52^ for discussion), which could further provide evolutionary drive to make decisions based on past costs. There are many cases where sunk costs can provide the additional incentive to push through difficult choices. For example, when pushing through the last part of a marathon run, a common coaching line is “you’ve come so far already, you can finish!” Similar incentives can be seen in the last segment of any long task that requires pushing through burnout and exhaustion, such as a PhD thesis. In truth, the correct comparison is between quitting (with no reward but no additional costs) and continuing (with the reward of finishing but with the additional costs of pushing through the burnout). It is possible that the costs of pushing through may appear over-burdensome because of economic myopia. The additional motivation provided by the refusal to quit (due to sunk costs) can allow one to achieve goals that may be difficult to achieve otherwise and may be linked to resilience. This motivational effect can be interpreted as the following logic: in a long marathon, there is uncertainty in that future valued goal. Thus it becomes easy to conclude that the goal is not worth the additional effort, particularly as that additional effort increases with exhaustion. But because the sunk costs provide additional evidence that the goal will be worth it, the past effort spent provides increased motivation.

## Methods

### Measures used

**Threshold** was found by a simple maximum-likelihood estimation over all possible thresholds from 0 s to 30 s. Value is defined as the difference between that threshold and the offered delay. (Thus an offer of 10 s with a threshold of 18 s provides a value of +8 s, while an offer of 20 s with a threshold of 18 s provides a value of −2s).

***p*(Earn)** was measured as the probability that an agent (mouse, rat, human, simulation) in a given (time-remaining, time-spent) pair remained to eventually earn the reward. Thus, if an agent has taken a 5 s offer, waited 2 s, it is in a [time-remaining = 3 s, time-spent = 2 s] pair. If the agent waited out the remaining 3 s to earn the reward on that offer, we counted that as an “earn”, while if the agent quit on the next second (after 3 s, with 1 s remaining), we counted that as a “not-earn”.

**The “sunk cost bubble”**. From the pEarn matrix, for each time-invested (time-spent), we summed the difference in probability of waiting out the delay (earning) between each time-remaining point and the probability of earning at that time-remaining point with 0 s invested. The measure was then the sum (over time-spent conditions) of those summed measures.

**Baseline slope**. We measured a linear fit to the probability of earning with 0 s invested. The measure was the slope of this fit line.

**Attrition bias**. The simulations provide **W0** values for each second that the agent is “in the wait zone” (i.e., took the deal, has not earned, and has not yet quit). We measured a linear fit to this scatter plot of these **W0** samples as a function of the time spent. The measure was the slope of this line.

### Simulations

Simulations were based on those of ref. ^35^. A simulated agent was designed that made two decisions: first an enter/skip decision (modeling the offer zone in Restaurant Row and WebSurf) and then a repeated quit/remain decision (modeling the wait zone) made once per second.

*Offer zone decision process*: In a given trial, the agent started with a draw of a willingness to wait, **W0** = **W**_**T**_ + **N(σ**_**W**_**)**, drawn from a normal distribution around the threshold (**W**_**T**_ = **T**_**OZ**_ defined as 18 s) with standard deviation **σ**_**W**_. If **W0** was greater than the threshold (**W0** ≥ **T**_**OZ**_), the agent accepted the offer and “entered the wait zone”.

*Wait zone decision process*: The willingness to wait **W(t)** drifted each second by adding in normally distributed random noise with standard deviation **σ**_**N**_. **W(t** + **1) = W(t) + N(σ**_**N**_**)** once per second. If **W(t)** decreased below the quit-threshold (**W(t) < T**_**WZ**_), the agent quit at time **t**. If the agent did not quit through the full delay, the agent “earned reward”.

The quit-threshold was defined as the linear function such that **T**_**WZ**_ = **W0** at the start of the waiting time and decreasing by 1 s each second until it reached **T**_**WZ**_ = 0 at the end of the waiting countdown. That is, **ΔT**_**WZ**_ = **–1/s**. If the cumulative willingness to wait (integrating the drift over time) ever fell below the quit-threshold TWZ, the agent “quit” out of the wait zone.

The **attrition bias** was measured as the distribution of initial **W0** willingness-to-wait that remained after a given time spent.

Trials were independent. The simulation did not do any learning and was assumed to follow the above decision process. Offers were uniformly distributed over 1s-30s inclusive. 1 million trials were run for each experiment.

Figure 4b–f: The initial parameter set used by Ott et al.^35^ was tested: **W**_**T**_ = **T**_**OZ**_ = 18 s. **σ**_**W**_ = 5 s. **σ**_**N**_.= 3 s.

Figure 4g–k: The offer zone decision process was changed to always accept the offer.

Figure 5: We did a parameter sweep running a full experiment (1 M samples evenly divided over 1s-30s offers inclusive) for each combination of (**σ**_**W**_ ∊ {0, 0.25, 0.5, 1, 3, 5, 8, 10, 20} x **σ**_**N**_ ∊ {0, 2, 3, 5}).

Figure 6a–c: The initial parameter set and the initial model as described above.

Figure 6d–f: **T**_**WZ**_ = **offer** and **ΔT**_**WZ**_ = **0/s**.

Figure 6g–i: **T**_**WZ**_ = **0** and **ΔT**_**WZ**_ = **0/s**.

Figure 6j–l: **T**_**WZ**_ was initialized to the offer and **ΔT**_**WZ**_ = + **1/s**.

Figure 7a–c: The initial parameter set and the initial model as described above.

Figure 7d–f: The modification used in Fig. 6d–f: **T**_**WZ**_ = **offer** and **ΔT**_**WZ**_ = **0/s**.

Figure 7g–i: The initial parameter set as described above, with the modification that we forced **W(t) ≤ W(t-1)**. If the random draw pulled **ΔW** > **0**, **W(t)** was set to be **W(t-1)**.

Figure 7j–l: Combining both modifications used in 7d, g.

Figure 8b, c: 20 simulations of 1 M offers each were made for each combination of (**σ**_**W**_ ∊ {0, 0.25, 0.5, 1, 3, 5, 8, 10, 20} x **σ**_**N**_ ∊ {0, 2, 3, 5}). The total sunk cost bubble and slope of the line at 0 s was measured as above. From each simulated session, we calculated the tangent of the probit fit at threshold and plotted the **σ**_**W**_ that generated it. The function was fit with MATLAB 2021a’s fit function (‘a + b/x’), adjusted-R2 as reported from the goodness-of-fit from that function. A hyperbolic fit was found (adjusted-*R*^2^ = 0.9997).

Figure 9: Simulations used the initial parameter set as described above, but agents accepted every offer and were prevented from leaving the wait zone for **DZ** seconds, after which **W(t)** was set to **W0** and drifted normally with standard deviation **σ**_**N**_. In the first model (9a), **T**_**WZ**_ was set to **offer** at the conclusion of **DZ** seconds and decreased at a rate of 1/s. In the second model (9e), **T**_**WZ**_ was set to **offer** at entry into the wait zone and decreased at a rate of 1/s as per the original model. Linear and quadratic fits were made with Matlab 2021a’s fit function, adjusted-R2 as reported from the goodness-of-fit from that function.

Figure 10: For rat and mouse data, latency to accrual was identified as the delay in seconds to when the sunk cost bubble began to grow. Outliers were defined as points with a latency to accrual larger than 15 s. For the rats, this included 97% of the data (all but one sample at 29 s, all other samples were in the range of 4s-8s). For the mice, this included 96% of the data (all but 5/115 samples). Linear and quadratic fits were made with Matlab 2021a’s fit function, adjusted-*R*^2^ as reported from the goodness-of-fit from that function.

### Subjects

Mice (Sweis et al.^19^) 32 M C57B6J mice, age 13 weeks

Mice (Durand-de Cuttoli et al.^20^) 32 M C57BL6J mice, age 10 weeks

Rats (Sweis et al.^19^) 4M 6F FBNF-1 rats.

Humans (Sweis et al.^19^) 65 undergraduate students were recruited from the University of Minnesota (24 M, 41 F, mean age = 20 yrs). Ethnicity as reported by the students: 73% white, 16.5% asian, 4.5% Black/African American, 2.5% Hispanic/Latinx, 3.5% other or declined to answer.

Humans (Huynh et al.^23^) 178 humans, including 31 undergraduates recruited from Wabash College, and the rest recruited online. Online recruited from Amazon mTurk, restricted to workers form the US. 97 M 80 F 1 non-binary as identied by the subject. Mean age = 39 yrs. Ethnicity as reported by the subject: 3% Black/African American, 62% white, 8.5% asian, 5.5% Hispanic/Latinx, 21% other or declined to answer.

Humans (Kazinka et al.^26^) 259 humans in the original version; 280 humans in the distractor version. Online recruited through Amazon mTurk. Inclusion criteria detailed in the original study^26^. Original version: 47% M, 53% F, <1% non-binary as reported by subject, mean age = 37 yrs; Distractor version: 50% M, 49% F, <1% non-binary as reported by subject, mean age = 38 yrs. Ethnicity as reported by the subject: Original version, 73% White, 10% Black/African American, 10% mixed/other, 7% Asian, 1% refused. Distractor version: 77% White, 12% Black/African American, 5% Asian, 4% mixed/other, 1% refused.

Humans (NCST dataset) 232 humans. Online recruited from Prolific, restricted to participants from the US. 110 M 117 F 5 non-binary as identified by the subject. Mean age 37. Ethnicity as defined by the subject: 9% Black/African American, 68.5% white, 7% asian, 8% Hispanic/Latinx, 7.5% other or declined to answer.

Rats (PJC/GWD dataset):7 M 8 F FBNF-1 rats, age 10–17 mos.

Rats (BJC/YAB dataset): 21 M BN rats, age 8–12 mos.

### Data reporting and ethics statements

Experimental data analyzed here were drawn from the following experiments:

Figure 3: **a** Mice, Sweis et al. 2018;^19^ **b** Mice, Durand-de Cuttoli et al. 2022;^20^ **c** Rats, Sweis et al. 2018;^19^ **d** Humans, (UG in person) Sweis et al. 2018;^19^ **e** Humans, (online) Huynh et al. 2021;^23^ **f** Humans, (online) [NCST dataset].

Figure 8: **d** Mice, Sweis et al. 2018^19^, Durand-de Cuttoli et al. 2022;^20^ **e** Rats, including Sweis et al. 2018^19^ and data from the Redish lab [PJC/GWD dataset].

Figure 10: **a** Rats without an offer zone from Sweis et al.^19^ **b**, **c** Rats, Sweis et al.^19^ and data from the Redish lab [BJS/YAB dataset]; **d** mice with limited experience, including mice C57BLJ6 from Sweis et al. 2018a,b^17,19^. **e**, **f** Each mouse provides two lines, one early and one late.

All experiments were approved by the appropriate boards and comply with the ethical regulations of their respective universities as well as US National Institute of Health guidelines. All human subjects provided informed consent as in the original studies and as follows:

- Approved by the University of Minnesota IACUC: Sweis et al. 2018^19^, PJC/GWD dataset, BJS/YAB dataset.
- Approved by the Mount Sinai IACUC: Durand-de Cuttoli et al. 2022^20^.
- Approved by the University of Minnesota IRB: Sweis et al. 2018^19^, Kazinka et al. 2021^26^.
- Approved by the Wabash College IRB: Huynh et al. 2021;^23^ NCST dataset.

All human studies were designed to include as diverse a population as possible. Inclusion criteria were described in the original studies and are provided above.

### Reporting summary

Further information on research design is available in the Nature Research Reporting Summary linked to this article.

## Supplementary information

Reporting summary

## Data Availability

Simulation code, data, and figure generation code are all available at https://github.com/adredish/SunkCostModelsAndData2022.
